# Photoluminescence Study of the Influence of Additive Ammonium Hydroxide in Hydrothermally Grown ZnO Nanowires

**DOI:** 10.1186/s11671-018-2665-4

**Published:** 2018-08-22

**Authors:** A. S. Dahiya, S. Boubenia, G. Franzo, G. Poulin-Vittrant, S. Mirabella, D. Alquier

**Affiliations:** 1 GREMAN UMR 7347 Université de Tours, CNRS, INSA Centre Val de Loire, 16 rue Pierre et Marie Curie, 37071 Tours CEDEX2, France; 2 MATIS IMM-CNR and Dipartimento di Fisica e Astronomia, Universita’ di Catania, via S. Sofia 64, 95123 Catania, Italy; 3 GREMAN UMR 7347 CNRS, Université de Tours, INSA Centre Val de Loire, 3 rue de la Chocolaterie, CS 23410, 41034 Blois CEDEX, France

**Keywords:** Zinc oxide, Nanowires, Hydrothermal, Photoluminescence, Ammonium hydroxide

## Abstract

We report the influence of ammonium hydroxide (NH_4_OH), as growth additive, on zinc oxide nanomaterial through the optical response obtained by photoluminescence (PL). A low-temperature hydrothermal process is employed for the growth of ZnO nanowires (NWs) on seedless Au surface. A more than two order of magnitude change in ZnO NW density is demonstrated via careful addition of NH_4_OH in the growth solution. Further, we show by systematic experimental study and PL characterization data that the addition of NH_4_OH can degrade the optical response of ZnO NWs produced. The increase of growth solution basicity with the addition of NH_4_OH may slowly degrade the optical response of NWs by slowly etching its surfaces, increasing the point defects in ZnO NWs. The present study demonstrates the importance of growth nutrients to obtain quality controlled density tunable ZnO NWs on seedless conducting substrates.

**Electronic supplementary material:**

The online version of this article (10.1186/s11671-018-2665-4) contains supplementary material, which is available to authorized users.

## Background

Significant developments in the synthesis of functional nanomaterials via bottom-up approaches are now offering high-quality materials for the development of next-generation efficient electronic devices [1–5]. The ZnO’s field of research has shown resurgence in interest after the successful demonstration of the growth of single-crystalline nanostructures (nanobelt) [6]. Thereafter, the use of high-quality, single-crystalline semiconducting ZnO nanostructures for the assembly of high-performance electronics continues to attract enormous research interest in the field of displays [7, 8], logic circuits [9, 10], sensors [11, 12], and optoelectronics [13]. The renewal of interest in ZnO material has largely been driven by its bio-compatibility, facile nanostructure fabrication, and large family of achievable nanomorphologies [14, 15]. Among the various different ZnO nanoarchitectures, one-dimensional (1D) ZnO nanowires (NWs) and nanorods (NRs) have been investigated comprehensively as an active semiconducting material in nanoscale devices such as field-effect transistors (FETs) [16], nanogenerators (NGs) [17], or sensors [12].

Ideally, a well stoichiometric ZnO is an insulator. However, in its non-stoichiometric form, it can behave as semiconductor or conductor depending on the number of native point defects created and/or by amount of dopant introduced. It has been shown that, in nanostructured ZnO, defects play a central role in defining the electronic device performances, as for sensors [18] and/or nanogenerators [17, 19], by controlling free charge density, minority carrier life time, and luminescence efficiency. For instance [18], it has been shown that highly sensitive UV sensors can be obtained by increasing the number of surface defects in ZnO NWs. These surface defects may act as trapping centers for free electrons and results in the formation of surface depletion layer. The greater the depth of depletion region at the NW surface, the higher the UV sensitivity. On the other hand, a too large number of defects have detrimental effects on the NG device performances [17, 19]. Therefore, a perfect control over the quality of ZnO nanomaterial produced is essential to build a high-performance electronic device.

Different bottom-up growth techniques, including flame transport approach [20–23], vapor-liquid-solid (VLS) [24], electrochemical deposition [25], and hydrothermal and/or chemical bath deposition [16, 26–29] have been utilized for the synthesis of 1D ZnO NWs. Nevertheless, most of the techniques are limited by their high-temperature process that cannot be scaled up over large device area at very low cost, on plastic substrates for example. The need of a facile, industrially scalable, and substrate-independent synthesis of ZnO NWs has seen significant advancements towards the hydrothermal growth process [16, 17]. Hydrothermal growth (HTG) is a low-temperature process where single-crystalline 1D material can be produced on various substrates, including plastics or even textile fibers [30]. In general, HTG-grown ZnO NWs show intense defect level band peak in photoemission spectra which expands from blue to red color wavelength emission depending on type of defects in the nanomaterial [31]. In the literature, many different point defects such as oxygen and zinc vacancies (V_O_ and V_Zn_) and interstitial (O_i_ and Zn_i_), antisites (O_Zn_ and Zn_O_), and hydrogen impurities were identified to be the cause of the defect level emission band in photoluminescence (PL) [32]. The visible PL band consists of three Gaussian components at 2.52, 2.23, and 2.03 eV, respectively labeled as blue I_B_, green I_G_, and orange I_O_ peak emission [33]. However, even after years of investigations, the origin of these defect states is still a subject of debate. Nevertheless, irrespective of the cause of defects in ZnO, the ratio of the band-to-band transition (UV emission) to the defect-related peak intensity in PL spectrum predicts the optical response of the nanomaterial produced [18, 34].

Growth process with an in situ integration of ZnO NWs over a metal electrode without any ZnO seed layer may improve the charge transport process across the metal-semiconductor (MS) contact interface and, in consequence, may improve device performances [35]. Ammonium hydroxide (NH_4_OH) has often been employed for the growth of ZnO NWs on Au metal surfaces [35, 36]. For instance, in our previous work, we show that NH_4_OH can be used for simultaneous tuning of the NW density and electrical properties of the ZnO NWs grown on seedless Au surface [5]. However, report detailing the effect of the addition of NH_4_OH over the optical response of the produced ZnO nanomaterial on Au surface is rarely found in literature. In the present report, we study the ZnO material optical response by analyzing the defect-related emission and UV emission in PL spectrum of NWs grown in different NH_4_OH concentrations. Two dominant peaks, noticed in the PL graph, are centered at 3.24 eV (382 nm) and 2.23 eV (556 nm), respectively referred as ultraviolet (UV) emission (I_UV_) and green defect level emission (I_G_) peaks. The extracted ratio I_UV_/I_G_ provides a qualitative index of the radiative defect amount in the produced nanomaterial. The effect of NH_4_OH is further confirmed by carrying out another series of experiments and PL characterizations. In this second series of experiments, we have grown ZnO NWs without NH_4_OH and, then, carried out a post-growth treatment of NWs in ammonia solution with different pH. We found out a similar trend of decrease in the ratio I_UV_/I_G_ for both series of samples, i.e., the ones grown in different NH_4_OH concentration and the other ones post-growth treated in NH_4_OH.

## Methods

The ZnO NWs are grown by hydrothermal growth process on (100) oriented Si wafers. A sample of 2 × 2 cm^2^ rigid silicon is first cleaned in piranha solution (1:1 H_2_SO_4_ and H_2_O_2_) for 10 min followed by a 2-min dip in hydrofluoric acid (50%) to remove the thin oxide formed during piranha cleaning and, finally, rinsing in deionized (DI) water. This cleaning step is followed by drying with nitrogen gas, and a final baking step is performed at ~ 200 °C to remove any adsorbed moisture before the metal deposition. A gold layer (~ 200 nm thick) is then deposited by direct current sputtering technique at room temperature. To improve the adhesion between gold and silicon, we deposit a layer of titanium (~ 100 nm) using the same technique. The reactant precursor for HTG consists of 1:1 ratio of zinc nitrate hexahydrate (Zn (NO_3_)_2_‚6H_2_O, 98% Sigma Aldrich) and hexamethylenetetramine (HMTA, Sigma Aldrich). During the growth, the substrates were immersed facing down in a Teflon cup, sealed inside stainless steel autoclave reactor and placed in a preheated convection oven at 85 °C for 15 h. The autoclave is taken out from the oven and cools down naturally. The substrates are then thoroughly rinsed with flowing DI water and dried in N_2_ gas flow. In the experiments, the concentration of NH_4_OH is varied from 0 to 50 mM. A Hitachi S-4150 scanning electron microscope (SEM) system is used for the morphological characterization of the ZnO NWs. To follow up the optical response of the obtained NWs with different NH_4_OH concentrations, photoluminescence (PL) measurements were performed; at room temperature (RT), by pumping at 1.5 mW, the 325 nm line of a He−Cd laser chopped through an acousto-optic modulator at a frequency of 55 Hz. Further experimental details for PL measurements can be found in Ref [33].

## Results and Discussions

**Fig. 1:**
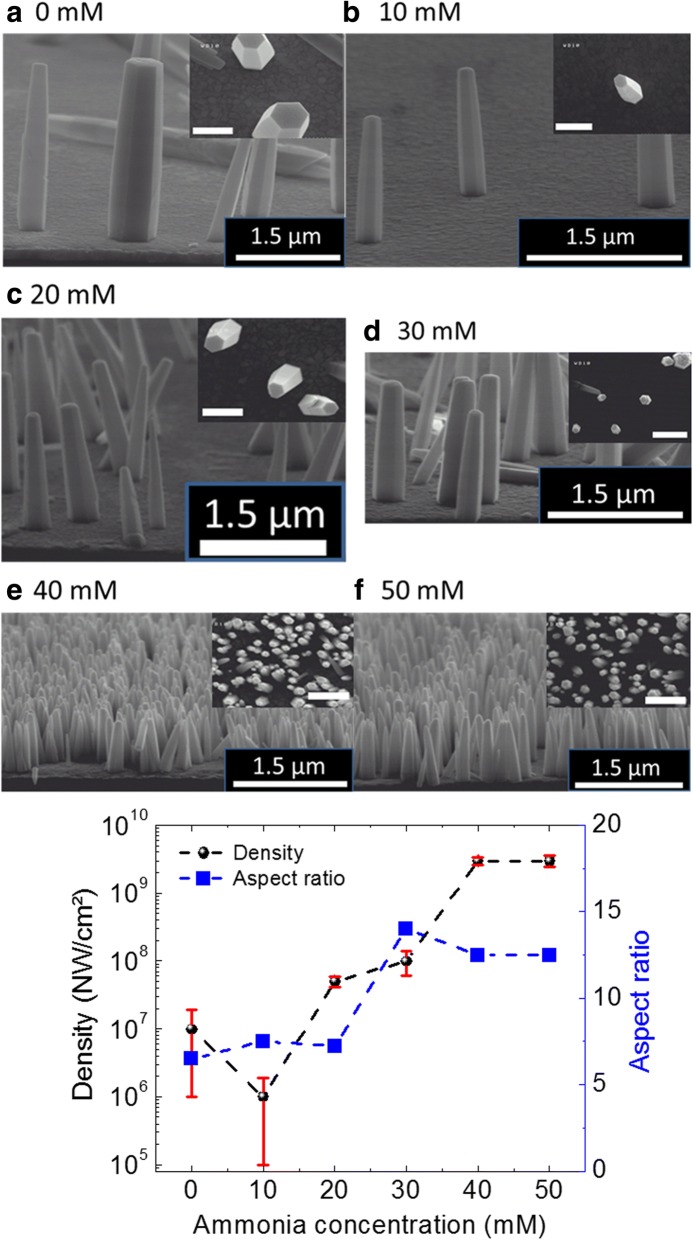
SEM images of NWs grown for different concentrations of ammonia. **a** 0 mM, **b** 10 mM, **c** 20 mM, **d** 30 mM, **e** 40 mM, and **f** 50 mM. The inset in each panel (**a**–**e**) shows the top-view SEM image acquired from the same sample. The scale bar in the inset is 500 nm. **g** The variation of density and aspect ratio of NWs with the change in NH_4_OH concentration

To execute the present study, ZnO NWs are grown using HTG process with different NH_4_OH concentration at 85 °C. The growth process parameters are mentioned in Table 1, and further growth details can be found in Ref. Boubenia et al. [5]. The obtained growth results while varying NH_4_OH concentrations (from 0 to 50 mM by step of 10 mM) in the growth solution are presented in Fig. 1a–f; showing typical cross-sectional and top-view SEM images acquired from the ZnO NW samples. A more than two orders of magnitude change in NW density is obtained by careful addition of NH_4_OH in the growth solution. The mechanism behind the NW density variation with NH_4_OH addition can be found in Boubenia et al. [5], where the authors hypothesized that the amount of ammonium hydroxide has a direct effect over the concentration of Zn (II) complexes which largely affects the Zn solubility in the solution. Consequently, the supersaturation of the growth solution can be controlled and so the number of nuclei over the substrate. Furthermore, going along with density, aspect ratio (AR) of the nanostructures greatly determines/conditions their application in flexible electronics where high surface to volume ratios are needed for increased strain absorption. Moreover, numbers of surface defect states are directly proportional to the AR of the NWs which has direct impact over the optical response of the nanomaterial. Hence, variation in NW’s AR, with increasing NH_4_OH concentration, is also calculated using SEM images. Figure 1g shows a graph depicting the density and AR variation trend with the addition of NH_4_OH in the growth solution. It can be seen, by using Fig. 1g, that, as the NH_4_OH concentration increases, both NW density and AR increase until the values saturate at ammonium hydroxide concentration of 40 mM. Room temperature Raman spectroscopy measurements, performed on ZnO NWs grown with different NH_4_OH concentrations, confirm the wurtzite crystal structure of the nanomaterial produced (Additional file 1: Figure S1) [5].

**Table 1:** HTG parameters for ZnO nanomaterial produced for each NH_4_OH concentrations at 85 °C

S. no.	Zinc nitrate and HMTA concentration (mM)	NH_4_OH concentration (mM)	pH	Growth time (h)
1	100	0	6.6	6
2	100	10	6.7	6
3	100	20	6.8	6
4	100	30	6.9	6
5	100	40	7.0	6
6	100	50	7.1	6

**Fig. 2:**
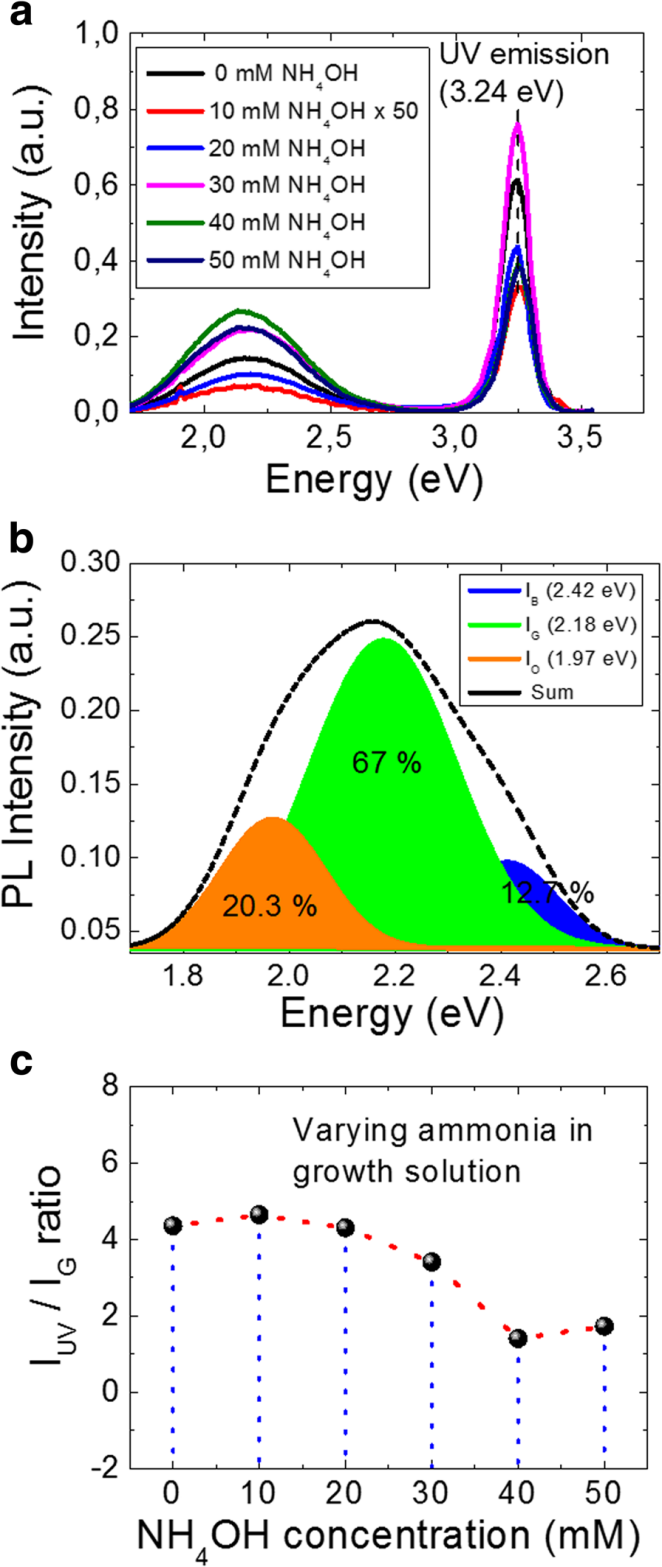
The PL measurements results. **a** The PL spectra of the ZnO NWs grown under different ammonium hydroxide concentrations. **b** Deconvoluted Gaussian fit for the 40-mM sample to blue, green, and orange emission states and their percentage contributions in visible emission. **c** The intensity ratio of UV and green emission peak as a function of NH_4_OH concentration

Figure 2a shows the PL spectra measured at RT for NWs grown with various ammonium hydroxide concentrations. The PL spectrum from ZnO NWs features two emission bands: a near-band-edge (NBE) light emission and a broad deep-level (visible) emission. The excitation energy used in the present study is 3.81 eV ensuring carrier population of the conduction band minimum. The strong and narrow UV emission peak, centered at 3.24 eV for all cases, results from merging of the various exciton-related emission near the band edge, including recombination of free excitons and its longitudinal optical (LO)-phonon replicas, [37] free-to-neutral acceptor transitions, [38] and donor-acceptor pair recombination [39], depending on the local lattice configuration and the presence of defects [40–42]. From Fig. 2a, we can also observe a broad visible level emission that expands from green to orange color wavelength. The presence of a broad visible emission peak may be explained with the hypothesis of existence of multiple defects and/or defect complexes which are dominantly present at the surface of ZnO nanostructures [34, 43]. However, in spite of a number of reports about the presence of visible emission in the ZnO’s PL spectrum, there is no clear consensus in the literature on the peak positions in the visible region or on their origin. It is also to note that, due to the large variation in density and aspect ratio from sample to sample (Fig. 1g), it is difficult to probe the same quantity of material for such different samples. Therefore, we cannot directly compare the emission intensity for these samples. Nevertheless, ratio of the magnitude of UV emission peak intensity with respect to defect-related peak intensity, in PL spectrum, predicts the optical response of the produced nanomaterial. All visible spectra can be successfully fitted by three defect-related visible luminescence states, namely blue, green, and orange. For instance, Fig. 2b plots the Gaussian fit of 40 mM NH_4_OH sample for the blue, green, and orange states, which are colored accordingly to emphasize their relative differences. It is to note here, although the PL intensity for both UV and visible emission peak varies due to difference in mass produced for varying NH_4_OH in solution, the percentage contribution for the blue, green, and orange states remains the same. In Fig. 2b, percentage contribution of each defect state, for the 40 mM sample, is presented, showing the major contribution of visible emission is related to the green defect state. Therefore, to follow up the optical response of nanomaterial produced, it is fair to compare the intensity ratio of UV emission (I_UV_) to green defect state (I_G_), which appears to have highest percentage contribution in the visible spectra.

Figure 2c plots the extracted I_UV_/I_G_ ratio for each case of NWs grown with different ammonia concentrations, whereas Table 2 presents the extracted values. It can be seen, using Fig. 2c, that the I_UV_/I_G_ ratio decreases smoothly after 20 mM of NH_4_OH in the growth solution. For instance, the I_UV_/I_G_ value for 40 mM ammonia is three times lower than the one of “no ammonia” sample, indicating an increase of visible defect states with the addition of ammonia. Dominantly, there can be two possible reasons for the observed experimental increase of visible level defect states: (i) increase of the aspect ratio and (ii) increase of the solution basicity with the addition of ammonium hydroxide. Considering the first point, aspect ratio can greatly affect the intensity of visible level emission. For instance, Djurisic et al. performed in-depth PL analysis on ZnO nanostructures and concluded that green luminescence in ZnO PL spectra probably originates from some non-paramagnetic defects or defect complexes and that the major part comes from surface defects [34]. It can be seen from Fig. 1g that there is a sharp increase of the aspect ratio (*L*/*d*) above 20 mM of NH_4_OH addition, where *L* and *d* are the length and diameter of the NW, respectively. A large AR signifies considerable increase of the surface to volume ratio, leading to enhanced defect level emission. Similar increase of the defect level emission with the increase of AR has been reported in Ref. [44] leading to a decrease in the I_UV_/I_vis_ ratio. However, the authors are not convinced that the increase of AR can be the sole reason for the observed increase in defect emission intensity. They further pointed out that the obtained results can be very sensitive to the experimental conditions such as excitation density and radiation area [44].

**Table 2:** Photoluminescence study of the influence of NH_4_OH addition over optical response of ZnO NWs

S. no.	NH_4_OH concentration (mM)	I_UV_	I_G_	I_UV_/I_G_	Free charge density (/cm^3^) [5]
1	0	0.61	0.14	4.4	4.3 ± 3.9 × 10^16^
2	10	0.33	0.07	4.6	N/A
3	20	0.43	0.1	4.3	8.3 ± 4 × 10^16^
4	30	0.75	0.22	3.4	N/A
5	40	0.38	0.27	1.4	2 ± 1 × 10^17^
6	50	0.38	0.22	1.7	N/A

Another possible reason to the observed increase in defect states in the NWs may be the addition of NH_4_OH itself. Chen et al. have shown that various defects can be induced in hydrothermally grown ZnO NWs (using ZnO seed layer) by the addition of NH_3_ molecules during the growth stage [45]. Although the addition of NH_4_OH is not crucial for the growth of ZnO NWs using ZnO seed layer, for the seedless growth of ZnO NWs on Au surfaces, the addition of NH_4_OH plays a key role in controlling the number of nucleation sites on Au surface. For example, Alenezi et al. explained the ZnO NW density variation on bare Au surface by stating that without NH_4_OH, mainly Zn^2+^ ions are available, while using ammonium hydroxide they are limited which significantly lowers the rate of homogeneous nucleation and encourages the heterogeneous one. Similar observations are reported by Boubenia et al. [5], where more than two orders of NW density can be varied by careful control of NH_4_OH concentration in the growth solution. Authors further claim an increase of free charge carrier density while field-effect mobility decreases as the NH_4_OH concentration increases, hinting the creation of extra point defects with the addition of NH_4_OH in the growth solution. However, no PL data is shown to confirm the reported electrical results. The PL results shown in Fig. 2 are in complete agreement with the electrical results reported by Boubenia et al. [5], as mentioned in Table 2, where the free charge density increases from 4.3 × 10^16^ to 2 × 10^17^ cm^− 3^ as NH_4_OH concentration increases from 0 to 40 mM in the growth solution. Therefore, we can hypothesize that the addition of NH_4_OH in the growth solution creates extra point defects in the ZnO NWs. Nevertheless, to confirm this hypothesis, we carried out another series of experiments where the as-grown ZnO NWs, without addition of NH_4_OH, are treated in solution with increasing basicity. The details of the post-growth treatment experiments are given in Table 3.

**Table 3:** Experimental parameters for the post-growth treatment of NWs in different concentrations of ammonium hydroxide solution and their effect over the optical response of the ZnO nanomaterial as measured by photoluminescence

S. no.	NH_4_OH concentration (mM)	pH	Time (min)	Temperature (°C)	I_UV_	I_G_	I_UV_/I_G_
1	As-grown	6.6	N/A	N/A	0.61	0.14	4.4
2	10	11.1	30	80	0.39	0.28	1.4
3	40	11.5	30	80	0.33	0.24	1.37
4	100	11.7	30	80	0.3	0.14	2.1
5	200	11.9	30	80	0.16	0.13	1.2

**Fig. 3:**
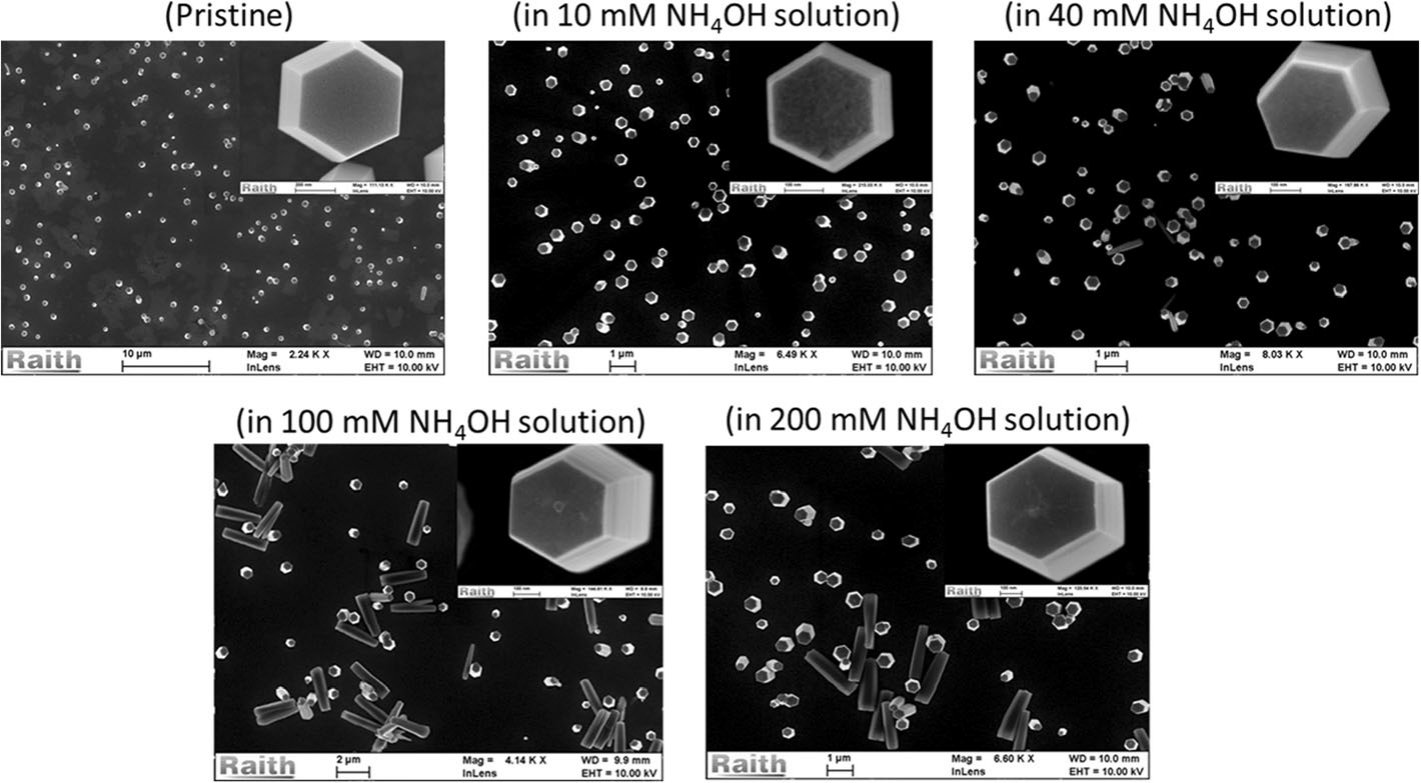
SEM images acquired from as-grown (pristine) ZnO NWs and post-growth treated NWs in different NH_4_OH concentrations

**Fig. 4:**
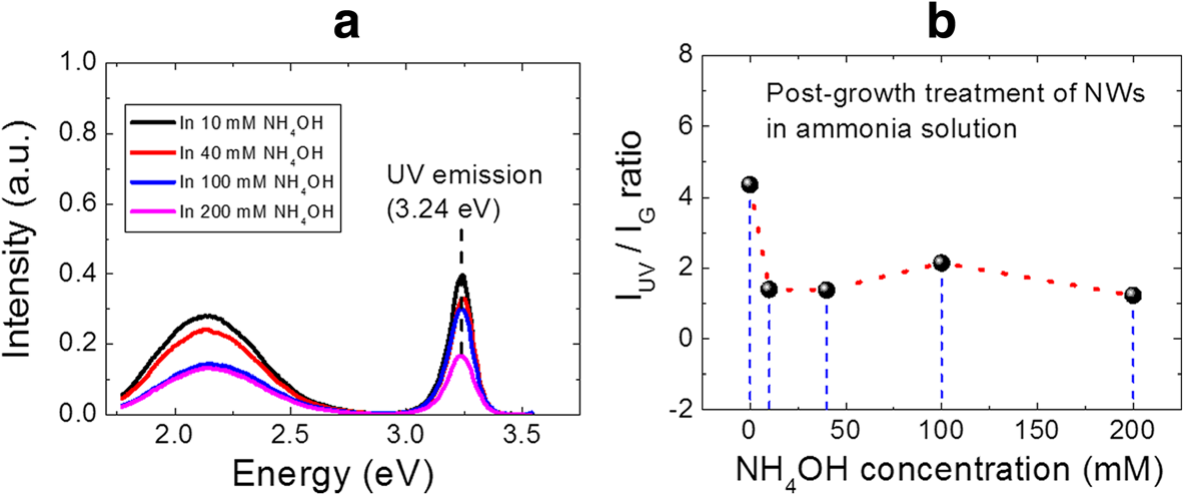
**a** The PL spectra of the ZnO NWs treated in solutions with various ammonium hydroxide concentrations. **b** The intensity ratio of UV and green emission peaks as a function of NH_4_OH concentration, as extracted from **a**

The obtained experimental results for post-growth treatment of NWs in ammonia solution are shown in Figs. 3 and 4. Figure 3 shows the corresponding SEM images of the samples treated in different NH_4_OH concentrations. It can be seen, from the present set of data, that the NW’s surface start to be rougher with increasing NH_4_OH concentration, even leading to the formation of nano-hillock at the polar surface of ZnO NWs for 100 and 200 mM treated samples. The worst case can be seen for samples treated with 100 and 200 mM NH_4_OH, where a few NWs seem to have broken from the base and are lying horizontally over the substrate. When further increasing the molarity of NH_4_OH solution, more than 90% of the NWs are broken (data not shown).

The resulting PL data arising from the post-growth treated samples are shown in Fig. 4. Figure 4a shows the PL spectra measured at RT for NWs treated with various ammonium hydroxide concentrations, whereas the extracted I_UV_/I_G_ plot is shown in Fig. 4b. It is to note that the peak position for both UV and visible emission has not been changed after NH_4_OH treatment, indicating that no extra point defect with different energy level is formed during the NH_4_OH treatment. The continuous reduction in the PL intensity of UV emission peak, with the increase of NH_4_OH concentration, clearly indicates the removal of ZnO nanomaterial due to a slow etching of the NWs in basic medium [46]. Furthermore, it is interesting to notice, from Fig. 4b, a clear and sharp decrease of the I_UV_/I_G_ ratio, as the NWs are treated in NH_4_OH solution. It is important to mention here that, for the present study, the experimental conditions such as excitation density, radiation area, initial mass of ZnO nanomaterial, etc. are fixed. Therefore, the observed I_UV_/I_G_ ratio trend can be entirely related to the effect introduced by the addition of NH_4_OH and not to changes in experimental conditions [47]. The obtained experimental results clearly support the hypothesis made in the previous section for creation of extra point defects with the addition of NH_4_OH in the growth solution. We believe that the increase of growth solution basicity with the addition of NH_4_OH can slowly degrade the optical response of NWs by slowly etching its surfaces, which increases the level of point defects in ZnO NWs.

## Conclusions

In summary, we demonstrated a facile, low-cost, and scalable bottom-up process for a seedless growth of ZnO NWs on metallic Au surfaces. With a careful addition of ammonium hydroxide in the growth solution, ZnO NW density can be controlled over two orders of magnitude. Consequences of the addition of NH_4_OH over the optical response of the obtained NWs were studied using photoluminescence technique. The visible emission spectrum, for each NH_4_OH concentration, was successfully deconvoluted to the blue, green, and orange defect states. Furthermore, percentage contribution of each defect state was also presented, showing the major contribution of visible emission was from green defect state. Thereby, to follow up the optical response of nanomaterial produced, we compared the intensity ratio of UV emission (I_UV_) to green defect state (I_G_). It was observed that the I_UV_/I_G_ ratio decreases sharply after the addition of 20 mM of NH_4_OH, hinting the creation of extra point defects with the addition of NH_4_OH in the growth solution. The experimental results were well supported by the literature data on the increase of free charge density with NH_4_OH addition. Nevertheless, the proposed hypothesis was further confirmed by performing another series of experiments where the as-grown ZnO NWs, without addition of NH_4_OH, were treated in solutions with increasing basicity. A clear and sharp decrease of the I_UV_/I_G_ ratio, as the NWs were treated in NH_4_OH solution, showed that the increase of growth solution basicity with the addition of NH_4_OH can slowly degrade the optical response of NWs by etching its surfaces which increases the level of point defects in ZnO NWs. The present study is important to control the optical response of ZnO NWs that can be directly grown on metallic Au electrodes for electronic and optoelectronic applications.

## Additional file

Additional file 1: **Figure S1.** Raman spectroscopy acquired from ZnO NWs grown with different ammonium hydroxide concentrations. (DOCX 99 kb)
