# Supplementary material for: Photoluminescence Study of the Influence of Additive Ammonium Hydroxide in Hydrothermally Grown ZnO Nanowires
Source: Nanoscale Res Lett. 2018 Aug 22;13:249. doi: 10.1186/s11671-018-2665-4 (PMC6104415; doi:10.1186/s11671-018-2665-4)
Supplement: Supplementary file 1 — Figure S1. Raman spectroscopy acquired from ZnO NWs grown with different ammonium hydroxide concentrations. (DOCX 99 kb) [file 11671_2018_2665_MOESM1_ESM.docx]

**Supporting Informations**

Photoluminescence study of the influence of additive ammonium hydroxide in hydrothermally grown ZnO nanowires

*A. S. Dahiya,^a^* S. Boubenia,^a^ G. Franzo,^b^ G. Poulin-Vittrant,^c^ S. Mirabella,^b^ D. Alquier^a^*

^a^GREMAN UMR 7347 Université de Tours, CNRS, INSA Centre Val de Loire, 16 rue Pierre et Marie Curie, 37071 TOURS Cedex2, France.

^b^MATIS IMM-CNR and Dipartimento di Fisica e Astronomia, Universita’ di Catania, via S. Sofia 64, 95123 Catania, Italy

^c^GREMAN UMR 7347 CNRS, Université de Tours, INSA Centre Val de Loire, 3 rue de la Chocolaterie, CS 23410, 41034 BLOIS Cedex, France.

*Corresponding author email: abhishek.dahiya@univ-tours.fr

**
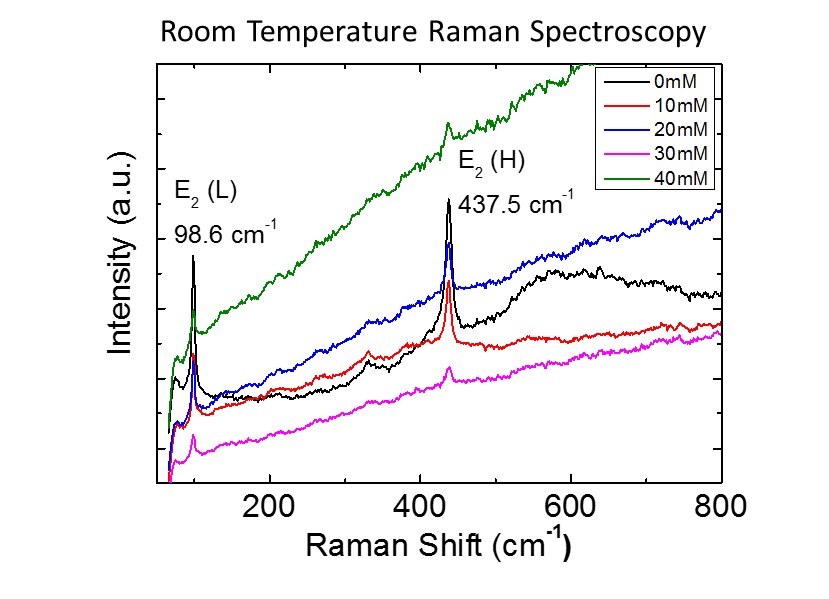
**

**Figure S1.** Raman spectroscopy acquired from ZnO NWs grown with different ammonium hydroxide concentrations.
